# Awareness, Perceived Importance and Implementation of Sports Vision Training

**DOI:** 10.3390/sports13100353

**Published:** 2025-10-04

**Authors:** Clara Martinez-Perez, Henrique Nascimento, Ana Roque, on behalf of the Sports Vision High-Performance Research Group

**Affiliations:** Instituto Superior de Educação e Ciências de Lisboa (ISEC Lisboa), Alameda das Linhas de Torres, 179, 1750-142 Lisboa, Portugal; henrique.nascimento@iseclisboa.pt (H.N.); ana.roque@iseclisboa.pt (A.R.)

**Keywords:** sports vision training, visual skills, coaches, awareness, implementation, reaction time, hand–eye coordination, perceived importance, visual performance

## Abstract

Background: Sports vision training improves perceptual–motor skills crucial for performance and injury prevention. Despite proven benefits, little is known about its perception and use among coaches in Portugal. Methods: A cross-sectional online survey was completed by active coaches from various sports, gathering sociodemographic data, awareness of visual training, perceived importance of ten visual skills, and implementation in training plans. Statistical analyses included descriptive tests to summarize sample characteristics, *t*-tests and two-way ANOVA to compare perceived importance of visual skills across sex and sport modalities, Spearman correlations to assess associations with age, and Firth-corrected logistic regression to identify predictors of incorporating visual training into practice plans. Results: Among 155 participants (88.5% men; mean age 36.9 ± 11.8 years), 73.2% reported incorporating visual training, with no association with self-reported knowledge (*p* = 0.413). Regarding perceived importance, reaction time was rated highest (1.20 ± 0.44), followed by hand–eye/body coordination (1.61 ± 0.71) and anticipation (1.34 ± 0.55). Age negatively correlated with importance given to visual memory, peripheral vision, concentration, depth perception, coordination, and moving-object recognition (*p* < 0.05). Multivariable analysis showed age (OR = 1.05; *p* = 0.0206) and volleyball (OR = 2.45; *p* = 0.031) positively associated with implementation, while higher perceived importance for visual concentration was negatively associated (OR = 0.54; *p* = 0.0176). Conclusions: Visual training implementation is high but not always linked to formal knowledge. Adoption is influenced by sport and demographics, and the counterintuitive role of visual concentration underscores the need for tailored educational programs to enhance performance and reduce injury risk.

## 1. Introduction

Sports vision training has emerged as a critical component for optimizing athletic performance across disciplines and competitive levels [1,2]. More than 80% of the sensory information athletes process during competition is visual, underscoring the central role of the visual system in environmental interpretation, decision-making, and technical execution [3].

Unlike static visual acuity alone, sports vision encompasses the ability to efficiently process, interpret, and respond to dynamic stimuli, integrating skills such as hand–eye and body coordination, peripheral vision, depth perception, reaction time, visual memory, concentration, contrast sensitivity, and moving-object recognition [4,5]. These skills are trainable, and improvements translate directly into competitive advantages: athletes with faster visual processing can anticipate plays earlier, react within fractions of a second, and make more accurate decisions under pressure [6].

Since the introduction of systematic ocular testing in U.S. basketball players in the 1960s, sports vision has evolved from an optional enhancement to an integrated element of physical and technical preparation [1]. Large-scale visual assessments during major events, such as the 1984 Los Angeles and 2004 Athens Olympic Games, highlighted a growing interest in quantifying and enhancing these abilities [1]. Institutions such as the *European Academy of Sport Vision* now define the field as a functional and multidisciplinary approach aimed at maximizing visual system performance according to the demands of each sport [4].

Welford’s information processing model describes three continuous phases, perception, decision, and execution, through which athletes process visual input [7,8]. First, sensory receptors capture external information; second, the brain selects and plans the appropriate response; third, motor commands are transmitted to execute the movement [9]. This cycle is dynamic: the perceptual system keeps receiving new input during movement execution, enabling real-time adjustments. This mechanism is crucial in interception sports such as cricket, baseball, or clay shooting, where milliseconds can determine the outcome [1,10].

Several studies have directly evaluated structured sports vision training protocols and their effects on both performance and injury prevention. Appelbaum and Erickson [2] showed that dynamic visual training improved batting performance in baseball players, while Clark et al. [3] demonstrated that vision training combined with reaction training reduced concussion incidence in collegiate American football. Krüger et al. [11] reported significant improvements in reaction time, coordination, and visual perception in cricket players after an eight-week visual skills training program, and Wilkins and Appelbaum [12] highlighted that peripheral vision training and perceptual–cognitive drills can enhance decision-making and situational awareness in team sports, with potential implications for reducing injury risk. Together, these studies highlight the dual role of sports vision training in enhancing perceptual–motor performance and contributing to injury prevention. Typically, sports vision training programs include exercises targeting hand–eye and body coordination, reaction time, peripheral awareness, depth perception, and concentration. More advanced protocols may also involve accommodative facility drills, dynamic visual acuity exercises, and the use of stroboscopic glasses to challenge visual–motor integration under time pressure [2,11].

Evidence consistently shows that targeted sports vision programs can significantly enhance reaction time, depth perception, accommodative flexibility, and hand–eye coordination [13,14]. These improvements have been observed across both precision sports, such as Olympic shooting, and invasion games like football and rugby [4,15]. Beyond performance gains, vision training can also improve attentional control, reduce visual fatigue, and support faster recovery from visual-motor errors during play [16].

Another benefit lies in the integration of visual and locomotor systems. During movement in complex environments, eye movements and gait patterns must be coordinated to maintain stability and minimize energy expenditure [17]. Studies have demonstrated that reduced stereopsis, such as through partial occlusion, impairs coordination and increases the risk of tripping, especially during high-obstacle navigation [18]. These findings extend to sports requiring rapid direction changes, jumps, or evasive maneuvers, where precise distance and height judgments are critical. Several controlled studies have demonstrated that sports vision training not only enhances perceptual–motor skills but also reduces injury risk. For example, improvements in visual-motor coordination and peripheral awareness have been associated with lower concussion rates in collegiate football and hockey [19,20], and with fewer musculoskeletal injuries in youth soccer and basketball [21,22]. Moreover, specific visual–motor interventions, such as dynamic vision training or strobe glasses, have shown effectiveness in reducing head impacts and lower-extremity injury rates in competitive athletes [2,3,23]

Correcting unaddressed refractive errors is equally important. Poor visual correction can impair visual information processing and, consequently, motor performance [24]. Contact lenses often offer advantages over spectacles in sports by providing a wider field of view, greater freedom of movement, and fewer disruptions during activity [25]. In aquatic or high-contact sports, however, careful consideration is needed to ensure safety and hygiene [26,27,28]. Active collaboration between coaches, sports medicine professionals, and vision specialists is essential to ensure that athletes receive optimal, sport-specific visual correction [29].

Given this evidence, sports vision training not only enhances perceptual–motor skills that directly impact competitive performance, but also contributes to injury prevention through improved spatial awareness, faster reaction capabilities, and more effective decision-making under pressure [3,5]. Despite these recognized benefits, little is known about how coaches across different sports perceive the relevance of specific visual skills or incorporate visual training into their practice routines. It should be noted that, in this study, the survey was designed to assess coaches’ general awareness and perceptions regarding sports vision training, rather than detailed knowledge of specific techniques (e.g., strobe training, accommodative facility drills, or dynamic visual acuity exercises). This study therefore aims to explore sports coaches’ awareness, perceived importance, and integration of visual skills training, providing empirical insights to guide targeted educational initiatives and the development of sport-specific vision training programs.

## 2. Materials and Methods

### 2.1. Study Design and Participants

A cross-sectional observational study was conducted with a convenience (non-probability) sample of sports coaches in Portugal. Recruitment took place between April and July 2024 through professional networks, sports clubs, and regional sports federations. Coaches represented a wide range of team and individual sports, including football, futsal, rugby, volleyball, rink hockey, padel, and several others with lower representation (e.g., basketball, swimming, gymnastics, tennis). The inclusion of multiple sport modalities was intended to provide a broad overview of awareness and implementation of sports vision training across different contexts. Inclusion criteria were: (1) being an active coach in any sport modality, (2) aged 14 years or older, and (3) providing informed consent to participate in the study. Coaches who provided incomplete responses for the primary variables of interest were excluded from the analysis. The study followed the ethical standards of the Declaration of Helsinki and was approved by the Ethics Committee of the Higher Institute of Education and Sciences of Lisbon (ISEC Lisbon) on 11 March 2024 (approval ID: CE/2024/03/11). The final sample consisted of 155 coaches (mean age 36.9 ± 11.8 years, range 14–63, median 37). The youngest 25% were aged 26 years or younger, while the oldest 25% were 46 years or older.

### 2.2. Instrument

Data were collected using an online structured questionnaire created in Google Forms, specifically designed by the authors for the purpose of this study, as no previously validated instrument was available in the literature to address the research questions. The questionnaire was developed in Portuguese, the native language of participants, and did not require translation. Its formulation was based on a review of existing literature on sports vision and visual skills [2,12,30,31], as well as consultation with experts in sports coaching and vision science to ensure content relevance. The instrument was composed of four sections:

- Sociodemographic and professional data: This section gathered information on participants’ age, gender, region of professional activity, sport modality, and athlete categories coached (e.g., youth, senior, elite). These variables allowed contextualizing the responses and stratifying analyses by demographic and sport-related characteristics. For clarity, definitions of all sport modalities included in the study are provided in Appendix A (Glossary of Sport Modalities).
- Awareness of Sports Vision Training: This part included items to determine whether participants had heard of sports vision training, their self-reported level of understanding, and whether they could define the concept. The aim was to assess baseline familiarity with the topic.
- Perceived importance of visual skills: Participants evaluated the importance of improving ten specific visual skills, hand–eye/body coordination, reaction time, anticipation, peripheral vision, visual memory, visual concentration, depth perception, recognition speed, contrast sensitivity, and moving-object recognition, for optimizing their athletes’ performance. Ratings were provided using a 5-point Likert scale, where 1 corresponded to very important and 5 to not important. This scale allowed quantifying the perceived relevance of each skill and facilitated statistical comparison between groups. As each Likert-scale item was intended to measure the perceived importance of a different visual skill, no factor analysis or internal consistency (e.g., Cronbach’s alpha) was conducted.
- Incorporation of visual training into practice plans: This section contained a dichotomous (yes/no) question regarding whether coaches currently integrate specific exercises to develop visual skills into their training sessions. Additional questions explored interest in receiving further information and whether their club or organization would be willing to invest in such training programs.

The questionnaire underwent face validation by a panel of three independent experts in sports vision and coaching. This validation process ensured that the wording, order, and content of the items were clear, relevant to the study objectives, and aligned with the practical realities of sports coaching. Minor adjustments were made based on the experts’ feedback to improve clarity and applicability. Formal reliability testing was not performed because each item was designed to capture a distinct visual skill rather than a unidimensional construct; therefore, expert review was considered the most appropriate method to ensure content validity in line with best practices [30]. A full copy of the questionnaire used in this study is provided in the Supplementary Materials (Supplementary File S1).

### 2.3. Data Processing and Statistical Analysis

Responses were exported from Google Forms and processed in R (version 4.4.2). Likert-scale responses were converted to numeric scores ranging from 1 to 5. Age was analyzed both as a continuous variable and as a categorical variable with five groups (≤25, 26–35, 36–45, 46–55, ≥56 years). Sport modalities were maintained according to their original categories, and those representing fewer than 3% of responses were aggregated into an “Other” category when appropriate. Statistical analyses were conducted in R using the packages dplyr, stringr, broom, car, emmeans, and ppcor. Descriptive statistics are presented as mean ± standard deviation (SD) for continuous variables and as absolute and relative frequencies for categorical variables. Independent-samples *t*-tests were applied for gender comparisons, and one-way ANOVA followed by Tukey post hoc tests was used to explore differences by sport modality. Correlations between visual skill scores and age were assessed using Spearman’s rank correlation. To examine the main effects and interaction between gender and sport modality for each visual skill, two-way ANOVA with Type III sums of squares was performed, setting contrasts to “contr.sum” for balanced interpretation. Spearman’s partial correlations between age and each visual skill were computed while controlling for sport modality. Associations between categorical variables (awareness and incorporation of visual training vs. gender, sport modality, and age group) were tested using Chi-square tests. When expected cell counts were below 5, *p*-values were estimated using Monte Carlo simulation with 10,000 replicates, a resampling approach that provides accurate significance levels when standard asymptotic assumptions are not met. A multivariable logistic regression model using Firth’s bias-reduction method was fitted to identify predictors of incorporating visual training into practice plans, including age, gender, sport modality, and standardized visual skill scores as independent variables, with odds ratios (OR) and 95% confidence intervals (CI) reported. Firth’s bias-reduction logistic regression was employed to address small cell counts and sparse data in certain categories, providing stable and unbiased parameter estimates compared to standard logistic regression models. A significance level of *p* < 0.05 was adopted for all analyses, and effect sizes such as Cohen’s *d* and η^2^ were calculated where relevant.

## 3. Results

### 3.1. Sample Characteristics

**Figure 1:**
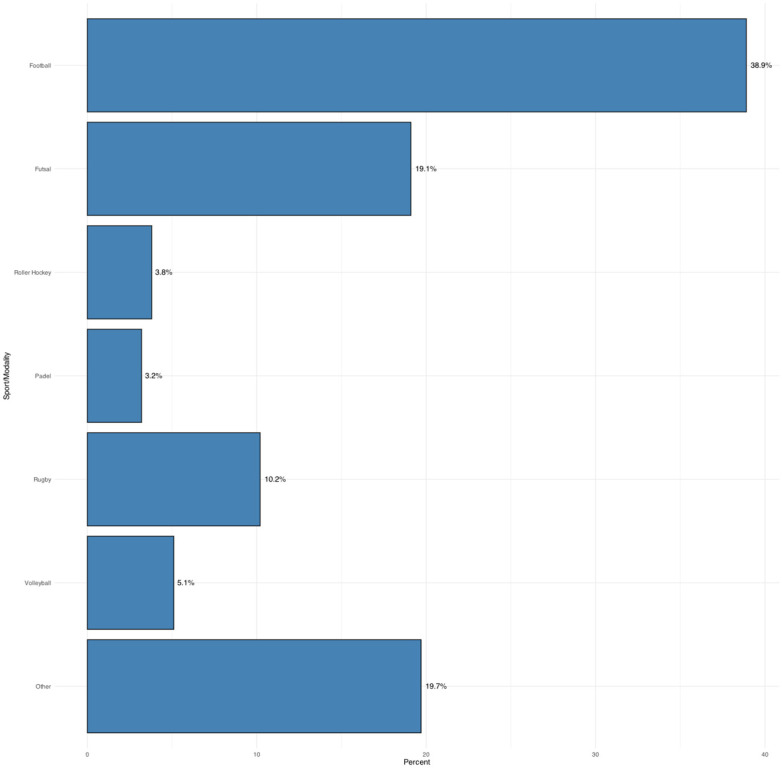
Distribution by Sport/Modality (%). “Other” includes the following modalities (each with fewer than five participants): Basketball, Gymnastics, Acrobatic Gymnastics, Judo, Swimming, Personal Trainer, Triathlon, Fitness, Football & Futsal, Golf, Motorsport, Swimming (Pure), Skateboarding, Water Ski & Wakeboard, Surfing, Laser Shooting & Fencing, Trampolining, and Tennis.

Of the 155 participants, most were men (88.5%) compared to women (11.5%). Regarding sport modality (Figure 1), the most frequent were football (38.9%), futsal (19.1%), and rugby (10.2%), followed by volleyball (5.1%), rink hockey (3.8%), and padel (3.2%). Other modalities, such as basketball, gymnastics, judo, swimming, triathlon, or individual sports like tennis and golf, showed lower frequencies (≤2.5%).

### 3.2. Stratified Comparisons (Sex, Age, Modality)

**Figure 2:**
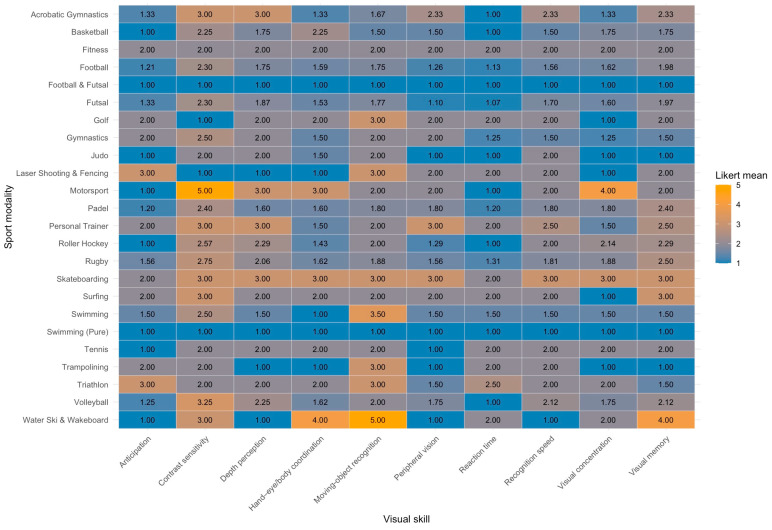
Mean importance by sport modality and visual skill.

Partial Spearman correlations between age and visual skills, controlling for modality, were negative for visual memory (ρₚ = –0.392, *p* < 0.001), peripheral vision (ρₚ = –0.257, *p* = 0.003), visual concentration (ρₚ = –0.216, *p* = 0.013), depth perception (ρₚ = –0.203, *p* = 0.020), hand–eye/body coordination (ρₚ = –0.191, *p* = 0.029), and moving-object recognition (ρₚ = –0.181, *p* = 0.038). A heatmap summarizing mean importance ratings for each visual skill across all sport modalities is presented in Figure 2. Two-way ANOVA (sex × modality) revealed that for hand–eye/body coordination, both sex (F = 9.42, *p* = 0.0028) and modality (F = 2.89, *p* = 0.012) effects were significant, but the interaction was not (F = 2.09, *p* = 0.061). Post hoc comparisons showed a significant sex difference only in basketball, with women assigning higher importance (+2.33 ± 0.76, *p* = 0.0028).

Table 1 presents the perceived importance (mean ± SD) of each visual skill by sport modality and sex.

#### 3.2.1. Visual Skills

The importance attributed to improving hand–eye/body coordination had a mean score of 1.61 ± 0.71 on the Likert scale. No statistically significant differences were observed according to sex (*p* = 0.7669), sport modality (*p* = 0.1639), or age (rho = –0.130, *p* = 0.1064).

Reaction time was rated the highest, with a mean of 1.20 ± 0.44. Significant differences were found by sex (*p* < 0.001), with the mean score in women being lower (1.00 ± 0.00) than in men (1.22 ± 0.47), indicating a higher perceived importance among women. 

Differences were also found by sport modality (*p* < 0.001). Tukey’s post hoc analysis showed that triathlon coaches gave lower relative importance than several modalities such as basketball, football, futsal, gymnastics, judo, padel, rugby, and volleyball, with differences ranging from 1.19 to 1.50 points on the scale. No significant differences were found by age (rho = –0.032, *p* = 0.6893).

For anticipation, the mean score was 1.34 ± 0.55. No significant differences were found by sex (*p* = 0.0606), but there were differences by modality (*p* < 0.001). The post hoc analysis again indicated that triathlon coaches scored lower than several modalities (basketball, football, futsal, acrobatic gymnastics, judo, padel, rugby, and volleyball), with differences ranging from 1.44 to 2.00 points. No significant differences were found by age (rho = –0.075, *p* = 0.3514).

#### 3.2.2. Visual Processing and Memory

Peripheral vision had a mean of 1.40 ± 0.63. No differences were detected by sex (*p* = 0.9280), but there were differences by modality (*p* < 0.001), with post hoc results indicating that the “Personal Training” modality had significantly higher scores than football (+1.74, *p* = 0.0062) and futsal (+1.90, *p* = 0.0017). In addition, a significant negative correlation with age was observed (rho = –0.282, *p* < 0.001), suggesting that the older the coach, the lower the perceived importance of this skill.

For visual memory, the mean was 2.04 ± 0.83. No differences were found by sex (*p* = 0.7978) or modality (*p* = 0.2336), but there was a significant negative correlation with age (rho = –0.382, *p* < 0.001).

Visual concentration had a mean of 1.67 ± 0.66. No significant differences were found by sex (*p* = 0.0691), but differences were found by modality (*p* = 0.0233). The post hoc analysis showed that the “Motorsport” modality gave higher scores than football, futsal, gymnastics, and judo (differences between +2.38 and +3.00 points). A negative correlation with age was also detected (rho = –0.173, *p* = 0.0311).

#### 3.2.3. Spatial Perception and Visual Discrimination

For depth perception, the mean was 1.90 ± 0.75. No differences were observed by sex (*p* = 0.2561) or modality (*p* = 0.1476), but there was a negative correlation with age (rho = –0.239, *p* = 0.0028).

For recognition speed, the mean was 1.72 ± 0.70, with no significant differences by sex (*p* = 0.3213), modality (*p* = 0.4626), or age (rho = 0.042, *p* = 0.6020).

Contrast sensitivity had a mean of 2.42 ± 0.92, the highest score among all skills (indicating the lowest perceived importance). No differences were found by sex (*p* = 0.4514) or modality (*p* = 0.0864), but there was a significant negative correlation with age (rho = –0.215, *p* = 0.0072).

For moving object recognition, the mean was 1.89 ± 0.88. No differences were found by sex (*p* = 0.0996), but there were differences by modality (*p* = 0.0394). The post hoc analysis indicated that “Water Skiing and Wakeboarding” had higher scores than basketball, football, and futsal (differences between +3.23 and +3.50 points). No significant differences were found by age (rho = –0.117, *p* = 0.1482).

#### 3.2.4. Incorporation of Visual Training into Practice Plans

A total of 73.2% of coaches reported including specific exercises for the development of visual skills in their training plans, while 26.8% did not. χ^2^ tests showed no significant differences by sex (*p* = 1.0000) or sport modality (*p* = 0.2139), but differences were found by age group (*p* = 0.0177). Specifically, the percentage of coaches incorporating these exercises progressively decreased with age: from 85.7% in those ≤25 years to 50.0% in those ≥56 years. Although coaches with greater awareness of sports vision training appeared more likely to implement it, no statistically significant association was found (*p* > 0.05).

### 3.3. Multivariable Logistic Regression

However, in a multivariable logistic regression (Firth’s bias-reduction) including age, sex, sport modality, and all visual-skill ratings, age was positively associated with including visual training (OR = 1.05 per year, 95% CI: 1.01–1.09, *p* = 0.0206; this OR reflects the adjusted cumulative effect of each additional year, independent of sport modality and the other variables included in the model), while higher importance given to visual concentration was negatively associated (OR = 0.54, 95% CI: 0.32–0.89, *p* = 0.0176). Volleyball coaches also showed higher odds (OR = 2.45, 95% CI: 1.09–5.48, *p* = 0.031). This reversal of the unadjusted age trend suggests confounding by modality and skill priorities, indicating that older coaches may be more likely to implement visual training when these factors are considered. Key outcomes from the model are summarized in Table 2.

## 4. Discussion

This study, the first in Portugal to address this topic, assessed the awareness, perceived importance, and implementation of visual skills training among coaches from different sport modalities. A total of 73.2% reported including specific exercises, although no statistically significant association was found between awareness and implementation (*p* = 0.413). This surprising finding suggests that coaches may implement visual training based on intuition, practical experience, or perceived benefits, rather than formal knowledge. In other words, adoption appears to be practice-driven rather than theory-driven, highlighting a gap that structured education programs could address. Reaction time emerged as the most highly valued skill, followed by hand–eye/body coordination and anticipation. This finding is consistent with prior evidence showing that reaction time and coordination are central to competitive success across multiple sports [2,12]. Age showed negative correlations with the importance attributed to visual memory, peripheral vision, visual concentration, depth perception, coordination, and moving-object recognition. Differences were identified across modalities and, to a lesser extent, by sex, while multivariable analysis revealed that age and volleyball were positively associated with implementation, whereas greater importance assigned to visual concentration was negatively associated. This unexpected negative association may reflect a potential mismatch between perceived complexity and practical feasibility. Coaches who assign high importance to visual concentration might regard it as a more abstract or difficult skill to operationalize within regular training routines, leading to lower practical implementation. Alternatively, they may perceive concentration as inherently linked to psychological or cognitive training rather than to specific visual drills, thus separating it from sports vision practice.

In terms of awareness of sports vision training, our results show that most coaches had heard of such programs, although the level of understanding and ability to define the concept varied considerably. This contrasts with findings by Dass et al. [32] in cricket and badminton coaches, where only 25% had heard the term “visual skills,” and no significant association was found between awareness and other variables. This difference may be attributable to the inclusion in our sample of sports such as football, futsal, and rugby, where the perceptual–motor component has received greater emphasis in coach training. Nevertheless, in both contexts, the need for educational programs to strengthen understanding and application of visual training remains evident. Similar calls have been made in broader sports medicine literature, where gaps in coach knowledge limit the application of evidence-based interventions [24]. Regarding sex differences, the finding that female coaches placed greater importance on reaction time aligns with patterns observed in other areas; for instance, Tsukahara et al. [33] found that female athletics coaches demonstrated greater awareness of specific medical issues, such as the female athlete triad (a syndrome involving low energy availability, menstrual dysfunction, and decreased bone mineral density), and engaged in more health-related discussions with their athletes than their male counterparts, suggesting these differences may be linked to a more holistic approach to performance and well-being. Additionally, recent work has shown that gender-related differences may also influence perceptual–cognitive learning processes, which could partly explain variations in how coaches value certain visual skills [34].

With respect to experience and age, our multivariable analysis revealed that, after adjusting for modality and skills, age was positively associated with implementation of visual training, a pattern distinct from that observed in the bivariate analysis and also from the findings of Anderson et al. [35] in school rugby, where experience did not necessarily translate into more structured injury management protocols. In our context, this may be due to veteran coaches in certain modalities, such as volleyball, integrating visual training more systematically. The high prioritization of reaction time, followed by hand–eye coordination and anticipation, is consistent with intervention studies by Coetzee and De Waal [36] and Krüger et al. [11], which reported significant improvements in these skills following targeted programs, as well as with the meta-analysis by Mann et al. [37], which concluded that expert athletes process perceptual cues more quickly and accurately than non-experts. This reinforces the idea that perceptual–cognitive skills differentiate expertise levels [37]. Importantly, the consistent prioritization of reaction time across modalities highlights its potential role as an anchor point for coach education initiatives. Positioning reaction time as an entry skill may facilitate the integration of broader visual training concepts into existing performance frameworks, providing a concrete starting point for practitioners less familiar with the field. Conversely, the lower valuation of peripheral vision among older coaches contrasts with findings from interventions such as those by Appelbaum et al. [16] and Clark et al. [20], where training improved peripheral perception and ocular tracking, indicating that, while this skill may not be prioritized by some coaches, its impact on performance is well documented. Similarly, visual memory and concentration showed negative correlations with age, suggesting that younger coaches tend to regard them as more relevant, a pattern not directly reflected in studies such as those by Dass et al. [32] or Tian et al. [38], but consistent with the idea that training priorities may vary by generation and initial coaching education.

Regarding the implementation of visual training, the 73.2% adoption rate observed in our study is markedly higher than the implicit figures in sports health and safety research, such as Van Hoye et al. [39], where health promotion was considered secondary to performance. This suggests that in Portugal there is a notable predisposition to integrate the visual component, even if not always underpinned by a deep formal knowledge base. The absence of a significant association between awareness and implementation (*p* = 0.413) is consistent with Dass et al. [32], who also found no correlation between self-reported awareness and practices, but differs from Tsukahara et al. [33], where greater knowledge translated into more concrete actions. Finally, although our cross-sectional data cannot prove causality, they support the importance attributed to reaction time, coordination, and ocular tracking, in line with improvements described in intervention studies by Coetzee and De Waal [36], Appelbaum et al. [16], and Krüger et al. [11]. The relatively low valuation of contrast sensitivity may be due to its lower visibility as a performance factor, despite evidence from Clark et al. [20] showing that training can enhance batting parameters, possibly through improved visual discrimination under variable lighting conditions. In broader contexts, perceptual vision training has also been shown to improve attentional control and decision-making beyond sport-specific settings, supporting its potential role in athlete development [40].

Our results also have implications for injury prevention. Coaches’ emphasis on reaction time and coordination is consistent with evidence that targeted visual–motor training can reduce concussion incidence and musculoskeletal injuries in youth and collegiate athletes [3,12,16]. However, the relatively low prioritization of peripheral vision and contrast sensitivity contrasts with studies showing their relevance for injury risk, particularly in contact sports [3]. Recent systematic reviews further confirm that sports vision interventions can play a dual role in enhancing both performance and safety outcomes [5]. In addition, anticipation and perceptual–cognitive training have been linked to a lower risk of concussions and improved injury resilience [41]. Together, these findings reinforce that visual training should not only be considered a performance-enhancing tool but also an evidence-based preventive strategy to improve athlete safety.

This study has some limitations that should be considered when interpreting the results. First, the cross-sectional design precludes establishing causal relationships between awareness, perceived importance, and implementation of visual training. Second, the sample was obtained through convenience sampling and included coaches from multiple modalities, predominantly football, futsal, and rugby, which may limit homogeneity and generalizability to other sports. Additionally, data were self-reported, introducing potential social desirability or recall bias. Although self-report bias cannot be ruled out, the clear trends observed—such as the universal prioritization of reaction time and the age-related decline in emphasis on peripheral vision and memory—suggest meaningful and consistent patterns in how visual skills are valued and integrated. These findings remain informative for guiding targeted interventions, even if future studies should validate them with objective assessments. Third, the questionnaire used was newly developed and, although reviewed by a panel of experts for content validity, no pilot study was performed and neither construct validity nor reliability (e.g., internal consistency, test–retest stability) were formally assessed. This limits the psychometric characterization of the instrument. Future research should therefore include pilot testing and apply robust methods to evaluate construct and convergent validity, as well as reliability, in order to strengthen the methodological rigor of subsequent investigations. Finally, the actual level of knowledge and the technical quality of the visual training programs implemented were not objectively assessed, which could have provided a more accurate understanding of the link between theory and practice.

Future research should employ longitudinal or intervention designs to evaluate changes over time and establish causal relationships. Including a larger and more balanced sample across sports and competition levels would enhance representativeness. It is also recommended to incorporate objective measurements of visual skills and technical quality of the programs implemented, as well as to explore barriers and facilitators to adoption using qualitative methods. Finally, comparing geographic and cultural contexts could provide insights into structural factors influencing the integration of visual training.

From a practical standpoint, our findings indicate that, although implementation rates are high, knowledge and precise definition of visual training remain variable, justifying the development of training programs specifically aimed at coaches. Such programs should be tailored to the specific demands of each sport, incorporate evidence on the effectiveness of different visual skills, and promote an integrated vision linking performance and injury prevention. Furthermore, raising awareness among clubs and federations about the benefits of visual training could facilitate its inclusion as a systematic component in physical and technical preparation plans.

## 5. Conclusions

Implementation of visual training among Portuguese coaches is relatively high, yet formal knowledge and structured education remain limited. Adoption appears to be influenced more by context and practice than by technical expertise. These findings underscore the need to develop evidence-based educational strategies tailored to sport-specific demands. Collaboration between sports federations, vision scientists, and coaching educators will be essential to design structured training modules that optimize athletic performance and contribute to injury prevention.

## Figures and Tables

**Table 1:** Perceived Importance of Visual Skills by Sport and Sex (Mean ± SD).

Sport	Sex	Anticipation	Contrast Sensitivity	Depth Perception	Hand–Eye/Body Coordination	Moving-Object Recognition	Peripheral Vision	Reaction Time	Recognition Speed	Visual Concentration	Visual Memory
Football	Female	1.00 ± NA	3.00 ± NA	2.00 ± NA	1.00 ± NA	2.00 ± NA	1.00 ± NA	1.00 ± NA	3.00 ± NA	2.00 ± NA	3.00 ± NA
Football	Male	1.22 ± 0.42	2.28 ± 0.87	1.75 ± 0.68	1.60 ± 0.62	1.75 ± 0.79	1.27 ± 0.52	1.13 ± 0.34	1.53 ± 0.65	1.62 ± 0.58	1.97 ± 0.76
Futsal	Female	1.20 ± 0.45	2.00 ± 0.71	2.00 ± 0.71	1.40 ± 0.55	1.20 ± 0.45	1.00 ± 0.00	1.00 ± 0.00	2.00 ± 0.71	1.40 ± 0.55	2.20 ± 0.84
Futsal	Male	1.36 ± 0.49	2.36 ± 0.76	1.84 ± 0.62	1.56 ± 0.77	1.88 ± 0.83	1.12 ± 0.33	1.08 ± 0.28	1.64 ± 0.57	1.64 ± 0.57	1.92 ± 0.76
Padel	Male	1.20 ± 0.45	2.40 ± 0.89	1.60 ± 0.55	1.60 ± 0.55	1.80 ± 0.45	1.80 ± 0.45	1.20 ± 0.45	1.80 ± 0.45	1.80 ± 0.45	2.40 ± 0.55
Roller Hockey	Female	1.00 ± 0.00	2.50 ± 0.71	2.50 ± 0.71	1.50 ± 0.71	2.00 ± 0.00	1.00 ± 0.00	1.00 ± 0.00	1.50 ± 0.71	2.00 ± 0.00	2.50 ± 0.71
Roller Hockey	Male	1.00 ± 0.00	2.75 ± 1.26	2.25 ± 1.26	1.50 ± 0.58	2.25 ± 1.26	1.50 ± 0.58	1.00 ± 0.00	2.50 ± 1.29	2.50 ± 1.29	2.25 ± 1.50
Rugby	Male	1.56 ± 0.63	2.75 ± 1.06	2.06 ± 1.00	1.62 ± 0.81	1.88 ± 1.02	1.56 ± 0.96	1.31 ± 0.48	1.81 ± 0.66	1.88 ± 0.81	2.50 ± 1.15
Volleyball	Female	1.33 ± 0.58	3.00 ± 0.00	2.33 ± 0.58	2.00 ± 1.00	2.00 ± 1.00	1.67 ± 0.58	1.00 ± 0.00	2.33 ± 0.58	1.33 ± 0.58	1.67 ± 0.58
Volleyball	Male	1.20 ± 0.45	3.40 ± 0.55	2.20 ± 0.45	1.40 ± 0.55	2.00 ± 1.00	1.80 ± 0.45	1.00 ± 0.00	2.00 ± 1.00	2.00 ± 0.71	2.40 ± 0.55
Other	Female	1.14 ± 0.38	2.00 ± 1.00	2.00 ± 1.15	1.57 ± 1.13	1.57 ± 0.79	1.71 ± 0.76	1.00 ± 0.00	1.57 ± 0.79	1.29 ± 0.49	1.71 ± 0.76
Other	Male	1.75 ± 0.79	2.46 ± 1.10	2.00 ± 0.78	1.79 ± 0.83	2.38 ± 1.01	1.75 ± 0.74	1.62 ± 0.71	1.88 ± 0.68	1.62 ± 0.77	1.92 ± 0.78

NA = Not applicable, used when there was only one respondent, and the standard deviation (SD) could not be calculated. “Other” includes sport modalities with fewer than five participants each, specifically: Basketball, Gymnastics, Acrobatic Gymnastics, Judo, Swimming, Personal Trainer, Triathlon, Fitness, Football & Futsal, Golf, Motorsport, Swimming (Pure), Skateboarding, Water Ski & Wakeboard, Surfing, Laser Shooting & Fencing, Trampolining, Tennis.

**Table 2:** Multivariable logistic regression model predicting inclusion of visual training.

Predictor *	Odds Ratio (OR)	95% CI (Lower–Upper)	*p*-Value
(Intercept)	13.59	1.00–237.83	0.0595
Age (per year)	1.05	1.01–1.09	0.0551
Male (vs. Female)	2.04	0.45–8.66	0.3376
Futsal (vs. Football)	0.67	0.21–2.15	0.4953
Rugby (vs. Football)	0.51	0.12–2.21	0.3628
Volleyball (vs. Football)	2.45	1.09–5.48	0.031
Other sports (vs. Football)	1.74	0.54–5.91	0.36
Eye–hand coordination	0.84	0.45–1.57	0.5916
Reaction time	0.35	0.10–1.09	0.0778
Anticipation	1.46	0.52–4.42	0.4838
Visual memory	1.41	0.60–3.42	0.4375
Visual attention	0.8	0.43–1.52	0.4895
Visual concentration	0.54	0.32–0.89	0.0226
Peripheral vision	1.2	0.53–2.72	0.6629
Visual reaction speed	0.71	0.33–1.53	0.3829
Visual acuity	0.81	0.42–1.55	0.5246
Visual tracking	0.9	0.49–1.67	0.7397

* Due to limited sample sizes, sport modalities with fewer than 10 cases (e.g., Padel, Roller Hockey) were grouped under the “Other” category to ensure model stability.

## Data Availability

The original contributions presented in this study are included in the article. Further inquiries can be directed to the corresponding author.

## Supplementary Materials

The following supporting information can be downloaded at: https://www.mdpi.com/article/10.3390/sports13100353/s1, Supplementary Material S1—Sports Vision Training Questionnaire.

## Appendix A. Glossary of Sport Modalities

**Acrobatic Gymnastics**: A gymnastics discipline combining tumbling, balance, and partner acrobatics.
**Basketball**: A team sport played on a court with the objective of scoring points by shooting a ball through a hoop.
**Fitness**: General training activities focused on strength, endurance, or conditioning, rather than a single competitive sport.
**Football (Soccer)**: The world’s most widely played team sport, in which two teams of 11 players attempt to score by moving a ball into the opponent’s goal.
**Football & Futsal**: Combined category including traditional football and futsal.
**Futsal**: A variant of soccer played indoors on a smaller court with five players per team.
**Golf**: An individual sport in which players hit a ball into a series of holes on a course in as few strokes as possible.
**Gymnastics**: Competitive artistic gymnastics, involving apparatus such as floor, vault, bars, and beam.
**Judo**: A martial art and Olympic combat sport emphasizing throws, holds, and grappling techniques.
**Laser Shooting & Fencing**: Precision sports involving either aiming with laser pistols or combat with fencing blades.
**Motorsport**: Competitive racing of motor vehicles, such as cars or motorcycles.
**Padel**: A racket sport similar to tennis, typically played in doubles on an enclosed court with glass walls.
**Personal Trainer**: Professional-led training sessions focusing on physical fitness and individualized conditioning.
**Roller Hockey (Rink Hockey)**: A team sport played on roller skates, using sticks to hit a ball (or puck) into the opponent’s goal.
**Rugby**: A contact team sport played with an oval ball, involving carrying, passing, kicking, and tackling.
**Skateboarding**: An action sport involving riding and performing tricks using a skateboard.
**Surfing**: A water sport where the athlete rides waves on a surfboard.
**Swimming**: Competitive swimming across various strokes (freestyle, backstroke, breaststroke, butterfly).
**Swimming (Pure)**: Subcategory indicating non-competitive or recreational swimming in pure form.
**Tennis**: A racket sport played individually or in pairs, where players hit a ball over a net into the opponent’s court.
**Trampolining**: A gymnastics discipline involving aerial acrobatics performed on a trampoline.
**Triathlon**: A multisport event combining swimming, cycling, and running in immediate succession.
**Volleyball**: A team sport where two teams of six players attempt to ground a ball on the opponent’s court over a net.
**Water Ski & Wakeboard**: Water sports involving being towed by a boat while gliding on skis or a board, often with acrobatics or jumps.
