# Supplementary material for: Awareness, Perceived Importance and Implementation of Sports Vision Training
Source: Sports (Basel). 2025 Oct 4;13(10):353. doi: 10.3390/sports13100353 (PMC12567578; doi:10.3390/sports13100353)
Supplement: Supplementary file 1 [file sports-13-00353-s001.zip › sports-3843389-supplementary.pdf]

Supplementary Material 1 – Sports Vision Training Questionnaire

Title:
Survey on Sports Vision Training among Coaches in Portugal

Purpose:
This survey was developed as part of the Postgraduate Program in Sports Vision Training at ISEC Lisboa, aiming to assess the level of knowledge about sports vision training in Portugal, specifically coaches' perceptions.

Instructions:
- All responses are anonymous and confidential.
- Completing the questionnaire takes approximately 5 to 10 minutes.
- *Items marked with an asterisk ()* are mandatory.*

Questionnaire

1. **Please indicate your age:** *[Open answer]*
2. **Please indicate your gender:**
    - Male
    - Female
3. **In which region of the country do you work as a coach?**
    - North
    - Center
    - West and Tagus Valley
    - Greater Lisbon
    - Setúbal Peninsula
    - Alentejo
    - Algarve
    - Azores
    - Madeira
    - None of the above. I coach outside Portugal.
4. **What is the sport(s) in which you coach?** *[Open answer]*
5. **What age category/categories do your athletes belong to?**
    - Under-8
    - Under-10
    - Under-12
    - Under-14
    - Under-16
    - Under-18
    - Seniors
6. **Have you ever heard about Sports Vision Training?**
    - No
    - Yes, but I am not familiar with the topic
    - Yes, I know exactly what it is
7. **Which Visual Skills do you consider essential for your athletes' performance?** *(Check all that apply)*
    - Eye–Hand / Eye–Body Coordination

- Reaction Speed
- Anticipation
- Peripheral Vision
- Visual Memory
- Visual Concentration
- Depth Perception
- Object Recognition Speed at Different Distances
- Contrast Sensitivity
- Ability to Recognize Moving Objects

8. **How important is it for you to improve Eye–Hand/Eye–Body Coordination in your athletes?**
    - 1 – Very Important
    - 2 – Important
    - 3 – Indifferent
    - 4 – Slightly Important
    - 5 – Not Important
9. **How important is it for you to improve your athletes' Reaction Time?**
    - 1 – Very Important
    - 2 – Important
    - 3 – Indifferent
    - 4 – Slightly Important
    - 5 – Not Important
10. **How important is it for you to improve Anticipation in your athletes?**
    - 1 – Very Important
    - 2 – Important
    - 3 – Indifferent
    - 4 – Slightly Important
    - 5 – Not Important
11. **How important is it for you to improve your athletes' Peripheral Vision?**
    - 1 – Very Important
    - 2 – Important
    - 3 – Indifferent
    - 4 – Slightly Important
    - 5 – Not Important
12. **How important is it for you to improve your athletes' Visual Memory?**
    - 1 – Very Important
    - 2 – Important
    - 3 – Indifferent
    - 4 – Slightly Important
    - 5 – Not Important
13. **How important is it for you to improve your athletes' Visual Concentration?**
    - 1 – Very Important
    - 2 – Important
    - 3 – Indifferent
    - 4 – Slightly Important
    - 5 – Not Important

14. **How important is it for you to improve your athletes’ Depth Perception?**
    - 1 – Very Important
    - 2 – Important
    - 3 – Indifferent
    - 4 – Slightly Important
    - 5 – Not Important
15. **How important is it for you to improve your athletes’ Object Recognition Speed?**
    - 1 – Very Important
    - 2 – Important
    - 3 – Indifferent
    - 4 – Slightly Important
    - 5 – Not Important
16. **How important is it for you to improve your athletes’ Contrast Sensitivity?**
    - 1 – Very Important
    - 2 – Important
    - 3 – Indifferent
    - 4 – Slightly Important
    - 5 – Not Important
17. **How important is it for you to improve your athletes’ ability to recognize moving objects?**
    - 1 – Very Important
    - 2 – Important
    - 3 – Indifferent
    - 4 – Slightly Important
    - 5 – Not Important
18. **Do you include specific exercises in your training plans to improve any of these visual skills?**
    - Yes
    - No
19. **Would you like to receive more information about Sports Vision Training specifically for your sport?**
    - Yes
    - No
20. **If yes to the previous question, please leave your email for contact:** *[Open answer]*
21. **In your opinion, would it be valuable for your athletes to benefit from Sports Vision Training as a complement to their regular training?**
    - Yes
    - No
22. **If yes to the previous question, would the club where you work as a coach be willing to invest in this service?**
    - Yes
    - No
    - Maybe
